# Distinctive biophysical features of human cell-types: insights from studies of neurosurgically resected brain tissue

**DOI:** 10.3389/fnsyn.2023.1250834

**Published:** 2023-10-04

**Authors:** Homeira Moradi Chameh, Madeleine Falby, Mandana Movahed, Keon Arbabi, Scott Rich, Liang Zhang, Jérémie Lefebvre, Shreejoy J. Tripathy, Maurizio De Pittà, Taufik A. Valiante

**Affiliations:** ^1^Division of Clinical and Computational Neuroscience, Krembil Brain Institute, University Health Network (UHN), Toronto, ON, Canada; ^2^Institute of Medical Science, University of Toronto, Toronto, ON, Canada; ^3^Krembil Centre for Neuroinformatics, Centre for Addiction and Mental Health, Toronto, ON, Canada; ^4^Neurosciences and Mental Health, The Hospital for Sick Children, Toronto, ON, Canada; ^5^Department of Biology, University of Ottawa, Ottawa, ON, Canada; ^6^Department of Mathematics, University of Toronto, Toronto, ON, Canada; ^7^Department of Psychiatry, University of Toronto, Toronto, ON, Canada; ^8^Department of Physiology, Temerty Faculty of Medicine, University of Toronto, Toronto, ON, Canada; ^9^Basque Center for Applied Mathematics, Bilbao, Spain; ^10^Faculty of Medicine, University of the Basque Country, Leioa, Spain; ^11^Institute of Biomedical Engineering, University of Toronto, Toronto, ON, Canada; ^12^Department of Electrical and Computer Engineering, University of Toronto, Toronto, ON, Canada; ^13^Division of Neurosurgery, Department of Surgery, University of Toronto, Toronto, ON, Canada; ^14^Center for Advancing Neurotechnological Innovation to Application (CRANIA), Toronto, ON, Canada; ^15^Max Planck-University of Toronto Center for Neural Science and Technology, University of Toronto, Toronto, ON, Canada

**Keywords:** human cortical tissue, rodent cortical tissue, epilepsy, pyramidal neurons, electrophysiology, morphology, transcriptomic, astrocyte

## Abstract

Electrophysiological characterization of live human tissue from epilepsy patients has been performed for many decades. Although initially these studies sought to understand the biophysical and synaptic changes associated with human epilepsy, recently, it has become the mainstay for exploring the distinctive biophysical and synaptic features of human cell-types. Both epochs of these human cellular electrophysiological explorations have faced criticism. Early studies revealed that cortical pyramidal neurons obtained from individuals with epilepsy appeared to function “normally” in comparison to neurons from non-epilepsy controls or neurons from other species and thus there was little to gain from the study of human neurons from epilepsy patients. On the other hand, contemporary studies are often questioned for the “normalcy” of the recorded neurons since they are derived from epilepsy patients. In this review, we discuss our current understanding of the distinct biophysical features of human cortical neurons and glia obtained from tissue removed from patients with epilepsy and tumors. We then explore the concept of within cell-type diversity and its loss (i.e., “neural homogenization”). We introduce neural homogenization to help reconcile the epileptogenicity of seemingly “normal” human cortical cells and circuits. We propose that there should be continued efforts to study cortical tissue from epilepsy patients in the quest to understand what makes human cell-types “human”.

## 1. Introduction

For decades, neuroscientific investigations have pursued the question: “What properties of the human brain allow for the uniqueness of the human experience?” With contributions of modern neuroscience, it is now understood that the quality of being human emerges from the interactions between the brain's 80 billion neurons. As inspired by the single neuron doctrine, resources from all over the world have been put toward understanding the constituent parts of the brain including individual neurons and their supporting glia (Yuste, 2015). Some institutions, such as the Allen Institute, have a dedicated mission to create atlases of the brain at various resolutions, consisting of macroscopic scale anatomical references to microscopic-scale molecular and cellular mapping (Hawrylycz et al., 2012; Han et al., 2022). These atlases provide an incredibly comprehensive evaluation of the brain at the structural level; however, they do not fully contextualize what functions the brain is performing over a temporal scale. While structural information can help inform how a neuron may function, based on channel expression, dendritic length, or spatial location, the structure/function relationship cannot necessarily discern a neuron's activity and how it may choose to interact within a neuronal network at a given time (Denk et al., 2012). As a result, the functional characterization of neurons and glia that make up the human brain, as well as understanding how they function relative to one another, remains a critical endeavor in experimental and computational neurosciences. However, limited access to live human brain tissue has impeded the understanding of human cellular and network physiology. In turn, investigations have employed preclinical animal models, closely related to humans, to better address many scientific questions about the human brain such as those concerned with neurological conditions.

The scientific study of neurological conditions (diseases, disorders, and injuries) have made significant advances with the valuable insight provided by preclinical models (Cavalheiro et al., 1982, 1983; Cook and Cruicher, 1986; Avoli and Olivier, 1989; Avoli et al., 1994, 2006; Bragin et al., 2002; Duty and Jenner, 2011; Asgari et al., 2014; Lang et al., 2014; Moradi-Chameh et al., 2014; Moradi Chameh et al., 2015; Shojaei et al., 2014; Isakson et al., 2018; King, 2018; Cela et al., 2019; Vashi and Justice, 2019; Ahmed Juvale and Che Has, 2020; Rusina et al., 2021; Whitebirch et al., 2022). While preclinical modeling has contributed to the understanding of basic mechanisms underlying the conditions that transform the central nervous system, the neuronal properties of preclinical models cannot be fully translated to those of humans, inherently limiting their usefulness in studying disease (Fisher et al., 2009; Shanks et al., 2009; Shineman et al., 2011; Galanopoulou et al., 2013; Simonato et al., 2014).

The surgical resection of living brain tissue has provided the opportunity for researchers to increase their understanding of the human-specific features of human cell-types (Deitcher et al., 1991; Zeng et al., 2012; Mohan et al., 2015; Beaulieu-Laroche et al., 2018, 2021; Kalmbach et al., 2018, 2021; Berg et al., 2021; Moradi Chameh et al., 2021; Planert et al., 2021; Rich et al., 2021). Very few institutions worldwide, which perform surgical resections for the management of epilepsy and the removal of tumors, have access to living brain tissue for such investigations. As a result, there are limited studies that fully characterize the neuronal biophysical properties of human tissue and address the species differences that exist relative to preclinical models. While interneuron subtypes play an essential role in mediating the function of human circuitry, the focus of this review will be on pyramidal neurons and the supporting role of astrocytes. We summarize the evolution of studies investigating human brain tissue, present the methods of evaluating neuronal biophysical properties of pyramidal neurons and astrocytes of human tissue, compare these findings to preclinical model counterparts, and provide evidence for why such studies reflect “on average” the biophysical properties of “normal” human pyramidal neurons.

## 2. A review of *in vitro* studies on resected human brain tissue for the investigation of epileptogenesis and functional characteristics of human cell-types

Epilepsy is a chronic neurological disorder characterized by the debilitating presence of recurrent spontaneous seizures. In clinical cases where antiseizure medications (ASMs) fail to provide seizure freedom, resective surgery is recommended (Engel, 1992). This elective surgical procedure consists of removing the epileptogenic focus, which may render the patient seizure free (Rosenow and Luders, 2021). The earliest documented case of epilepsy surgery was performed in 1886 when scar tissue was removed from the cortex of the patient, leading to the complete remission of their seizures (Schijns et al., 2015), with diagnostic and surgical approaches refined by the pioneering work at the Montreal Neurological Institute by Wilder Penfield and Herbert Jasper. Since then, resective surgery has become a common clinical practice for the management of epilepsy.

Often, particularly in the context of mesial temporal lobe epilepsy (mTLE), brain tissue from the middle temporal gyrus (MTG) is removed to access the epileptogenic zone (Mansouri et al., 2012). In 1976, the first intracellular *in vitro* recordings were performed on human cortical tissue resected from a patient with epilepsy (Schwartzkroin and Prince, 1976), paving the way for additional laboratories to use resected tissue for characterizing human-specific neuronal biophysical properties. These initial investigations were primarily concerned with determining the appropriate control tissue to compare against the cortical tissue resected from patients with epilepsy. This debate produced three potential courses of action: using resected post-mortem tissue, “normal” cortical tissue from patients with epilepsy (removed from the MTG, an area that is farther away from the epileptogenic focus), or “normal” cortical tissue removed during tumor resections that is considered heathy, non-epileptogenic.

In theory, post-mortem tissue is the only source of “healthy” tissue, yet its poor quality can make it unsuitable for comparisons to living brain tissue. Due to conditions related to death, the post-mortem time interval, and the preservation methods, post-mortem tissue is often excluded from studies of neuronal function and may show genetic, molecular, and/or anatomical abnormal findings (Stan et al., 2006; Kramvis et al., 2018). However, the normality of cortical tissue removed from epilepsy patients remains a question, even when the tissue is seemingly “non-epileptogenic” (Schwartzkroin and Prince, 1976; Köhling and Avoli, 2006). As a result, the most reliable choice for control tissue consists of “normal” cortical tissue removed during tumor resections. However, this comparison does not solve the issue of functional differences in brain tissue that may arise from the use of different medications, the location of resection, and natural person-to-person variability, which may all contribute to the alterations of neuronal biophysical properties within human tissue. Beginning in the 1980s, rodents became the most advantageous and default choice for preclinical epilepsy models in research settings (Grone and Baraban, 2015). In turn, a comprehensive literature regarding the neuronal biophysical properties of preclinical rodent models of epilepsy exists that have been compared to human tissue (Avoli and Olivier, 1989; Avoli, 1993; Avoli et al., 1994). Despite efforts to control for age and location, it is clear there are fundamental differences between rodent and human neurons that inherently limit clinical translatability between the two species.

By necessity, however, these early studies defaulted to rodent tissue as control to compare against cortical tissue resected from patients with epilepsy, with a view toward characterizing the hypothesized aberrant electrical properties of human neurons engaging in epileptiform activity. In line with the single neuron doctrine (Yuste, 2015), the “epileptic neuron” (Schmidt et al., 1956; Ward et al., 1956; Ward, 2010) was a dominant concept and undergird for the *in-vitro* search for neurons displaying intrinsic action potential bursts and paroxysmal depolarizations, which would explain epileptiform synchronizations seen in patients (Schwartzkroin and Haglund, 1986; Avoli and Olivier, 1989; Avoli et al., 2006). Surprisingly, *in vitro* experiments revealed that cortical and hippocampal neurons from human epileptogenic tissue did not demonstrate significant changes in intrinsic properties when compared to healthy, non-epileptogenic neurons in controls (Schwartzkroin and Prince, 1976; Schwartzkroin and Haglund, 1986; Avoli and Olivier, 1989; Avoli, 1993; Telfeian et al., 1999; Mohan et al., 2015). Furthermore, when examining cortical tissue from patients demonstrating seizures, both near [frontal cortex from focal cortical dysplasia (FCD)] and far (MTG from temporal lobe epilepsy) from the seizure focus, a similar lack of change in the intrinsic properties such as afterhyperpolarization, adaptation index and firing pattern, resting membrane potential, input resistance and action potential amplitude of neurons was established (Avoli and Olivier, 1989; Foehring and Waters, 1991; Foehring et al., 1991; Avoli et al., 1994). However, other neuronal properties such as morphology, bursting, and convulsant sensitivity demonstrated changes with respect to the proximity to the epileptogenic focus (Avoli et al., 2005). For instance, it has been observed that the neurons in the frontal cortex show increased morphological changes as compared to neurons in the MTG that are more removed from the epileptogenic focus (Tassi, 2002). The frontal cortical neurons in patients with FCD also demonstrate higher bursting of action potentials and an increased sensitivity to the *in vitro* application of convulsants than tissue resected from the MTG of patients with epilepsy (Mattia et al., 1995). In addition, these neurons demonstrate obvious synaptic plasticity changes that decrease the inhibitory postsynaptic current and increase the excitatory postsynaptic current (Mattia et al., 1995; Avoli et al., 1999; Barbarosie et al., 2002). Conversely, *in vitro* studies on human and rat epileptic tissue have demonstrated an upsurge in asynchronous GABA release at both autaptic and fast-spiking-pyramidal neuron synaptic connections. This phenomenon leads to the desynchronization of fast-spiking- pyramidal neuron activities, playing a crucial role in mitigating runaway excitation and dampening the generation and propagation of epileptiform activities (Jiang et al., 2012). Overall, these studies did not demonstrate significant alterations in intrinsic properties through comparisons between MTG and FCD tissue as well as to healthy, non-epileptogenic neurons in control samples (Avoli and Olivier, 1989; Knowles et al., 1992).

As a result, investigations began to reorient their attention and explore other mechanisms underlying seizures and epileptogenesis, such as decreased excitation-inhibition balance or underlying structural changes that may be associated with increased excitability (Avoli and Olivier, 1989). However, most studies have failed to provide evidence of significant increases in excitability of cortical neurons from epileptogenic human tissue (Avoli and Olivier, 1989; Avoli and Williamson, 1996; Avoli et al., 2005, 2006). Therefore, if “epileptiform activity” is defined as abnormal discharges characterized by hypersynchronous and hyperexcitable activity, this type of “epileptiform activity” has not been shown in cortical or hippocampal neurons of tissue resected from patients with epilepsy. Consequently, the lack of conclusive evidence demonstrating that disease and medical treatments alter the basic intrinsic properties of cortical neurons remains a major concern in studies of human tissue.

## 3. Investigating the biophysical properties of the neurons of the human cortex through transcriptomic, morphological, and electrophysiological techniques: a comparative analysis between species

Chronologically, morphology was the earliest approach to cell-type classification, typified by the work of Cajal's which set in motion the single neuron doctrine. Such cataloging and classification of the unique structural features of brain neurons into cell-types, has been a long standing neuroscience endeavor to generate a parts list of the brain to infer function (Denk et al., 2012). An early example of such a structuralist approach was the profound inferences made about phase sequences, and the concepts of plasticity that Donald Hebb conjectured from the magnificent drawings of Cajal and Lorente de Nó (Hebb, 2005). Electrophysiological recording techniques like sharp electrode recordings and whole-cell patch clamping brought in the opportunity to functionally characterize single neurons and small circuits, complementing morphological classification, with a classification of electrical waveforms (Migliore and Shepherd, 2005; Ascoli et al., 2008). Such studies provided some clear associations between structure and function. For example, basket cells (i.e., somatic targeting somatic parvalbumin positive interneurons) have narrow spikes, and high firing rates, and distinct morphological features. However, this example is more of an exception, as it has become increasingly clear the use of single nucleus RNA sequencing (snRNA-seq), and patch-seq (Tasic et al., 2018; Scala et al., 2021) that a neuron's electrophysiological properties are only loosely associated with its morphology and transcriptome.

In this section, each technique will be introduced and its involvement in evaluating the neuronal biophysical properties of human tissue will be discussed. Importantly, while these techniques are inherently different in terms of mechanism and deliverables, they have all revealed species-specific differences in neuronal structure and function and shed light on the inherent cellular heterogeneity that exists in all species.

### 3.1. Morphological techniques highlighting the diversity of human cortical neurons

Morphological studies on tissue resected from the human cortex provide important information regarding the structural features of distinct neuronal types and how these features may impart a specific function within the microcircuit it is embedded in. Each neuron has the capacity to perform complex computations which are reliant on its relative dendritic and axonal structure. Various 3D constructions of pyramidal neurons of resected human tissue have revealed great heterogeneity across and within cortical layers (Deitcher et al., 1991; Mohan et al., 2015; Beaulieu-Laroche et al., 2018; Kalmbach et al., 2018, 2021; Berg et al., 2021; Moradi Chameh et al., 2021). As a function of depth, extending from the pia to layer 6 (L6), human cortical neurons exhibit increases in morphological complexity, arising from their size, dendritic arborization, and cellular density that are unparalleled in the rodent cortex (Deitcher et al., 1991; Mohan et al., 2015; Kalmbach et al., 2018, 2021; Berg et al., 2021; Moradi Chameh et al., 2021). Importantly, these structural findings were shown to be unrelated to disease etiology, severity, or duration (Mohan et al., 2015), suggesting these patient specific factors did not impact the morphology of human cortical neurons resected from epilepsy patients.

**Figure 1:**
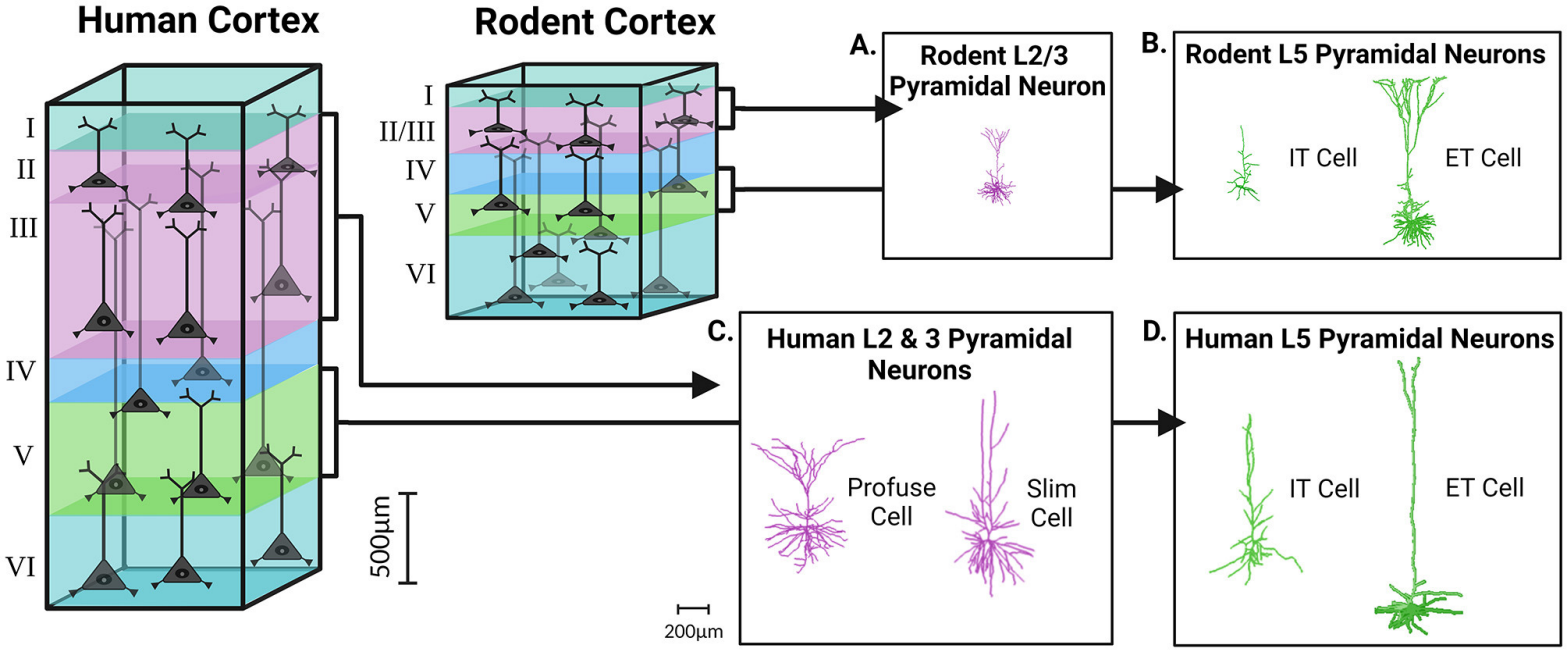
Cortical expansion in size and morphological complexity of pyramidal neurons in the human brain, as compared to the rodent. The rodent cortex, as compared to the human cortex, demonstrates a prominent reduction in overall cortical depth and laminar thickness. In rodents the boundary between L2/3 is indiscriminate, while all the layers of the human cortex are clearly demarcated from one another. 3D reconstructions of pyramidal neurons in the human, as compared to the rodent, display increases in morphological complexity, heterogeneity, and size within cortical layers. Pyramidal neurons of L2 and L3 of the human cortex can be subdivided into slim-tufted and profuse-tufted **(C)** unlike their rodent counterparts **(A)**. In both the rodent and human cortex, L5 can also be subdivided into extra-telencephalic (ET) or intra-telencephalic (IT) **(B, D)**. Morphological reconstructions for the human slim, profuse, and IT cells were obtained from our previous publication in Nature Communications (Moradi Chameh et al., 2021). The other reconstructions were obtained from NeuroMorpho.org including the human L5 ET cell (Akram et al., 2018; Shao et al., 2022), the rodent L2/3 pyramidal neuron (Boudewijns et al., 2013; Akram et al., 2018), the rodent L5 ET cell (Boudewijns et al., 2013; Akram et al., 2018), and the rodent L5 IT cell (Akram et al., 2018; Scala et al., 2021). Created with BioRender.com.

When comparing the morphological properties of cortical neurons obtained from human tissue to those in well characterized preclinical rodent models, many species-related differences were identified (Mohan et al., 2015; Berg et al., 2020). As shown in transcriptomic studies, human cortical layer 2 (L2) and layer 3 (L3) have well-defined boundaries unlike the homogeneous presentation of rodent L2/3 which are practically indistinguishable (DeFelipe, 2011; Markram et al., 2015; Gouwens et al., 2019) (Figure 1). These transcriptomic findings have been further confirmed by morphological studies showing that human cortical pyramidal neurons in L2 and L3 can be subdivided based on their dendritic morphology: slim-tufted, which has a low density of tuft branches, and profuse-tufted, which has a high density of tuft branches (Deitcher et al., 1991). These two cells show no depth separation in L2 and L3 but have varying biophysical properties that underlie functional differences (Deitcher et al., 1991). Interestingly, there is no existing homologous equivalent of slim-tufted and profuse-tufted cells within the rodent L2/3 cortex (Deitcher et al., 1991). When performing analysis on morphological data, one study found that 88% of human L2 and L3 pyramidal neurons can be grouped into human-specific clusters based on limited shared features with other L2/3 neurons from species such as mice and macaque monkeys (Mohan et al., 2015). In addition, the dendritic architecture of the L2 and L3 pyramidal neurons of humans, as compared to L2/3 pyramidal neurons of mice, demonstrate increased length (three times larger) and advanced arborization (Mohan et al., 2015). These findings reflect the uniqueness of slim- and profuse-tufted neurons to the L2 and L3 of the human cortex (Figures 1A, C).

Similar to L2 and L3, human layer 5 (L5) cortical neurons can also be subdivided with respect to their long-range axonal projection targets: extra-telencephalic (ET), which project to areas within and outside the telencephalon, or intra-telencephalic (IT) which have projections that are restricted to the telencephalon (Baker et al., 2018). Unlike slim- and profuse-tufted neurons, both ET and IT cells are found in the L5 cortical layer of mice yet exhibit species-related differences (Kalmbach et al., 2021) (Figures 1B, D). One of the more prominent species-related differences between L5 of human and rodent tissue is the abundance and distribution of ET cells. In the human cortex, ET cells are relatively scarce and only account for 2–6% of all excitatory neurons in L5. In contrast, ET cells in mice are much more abundant, comprising 20–30% of all L5 excitatory neurons (Hodge et al., 2019; Kalmbach et al., 2021). Despite these discrepancies in abundance, the morpho-electric properties and gene expression profiles of ET cells in humans are just as distinctive as those of their rodent counterparts (Beaulieu-Laroche et al., 2018, 2021; Kalmbach et al., 2021). The exact proportions of ET cells within L5 of resections of human tissue is subject to natural variability between patients (Hodge et al., 2019; Kalmbach et al., 2021). With respect to dendritic length, the apical dendrites of ET cells extend from L5 to the pia, however human cortical L5 IT cells have dendrites that extend past L3 (Baker et al., 2018; Beaulieu-Laroche et al., 2018; Kalmbach et al., 2021). Originally, this finding sparked much debate about whether or not the dendrites of IT cells were being unintentionally truncated during human cortical slice preparation.

Human cortical neurons are not simply scaled up compared to other species, but they also exhibit more complex dendritic arborization, with each branch capable of functioning independently (Deitcher et al., 1991; DeFelipe et al., 2002; Mohan et al., 2015; Gidon et al., 2020). This enhanced dendritic arborization allows human cortical neurons to make more extensive and widespread synaptic contacts with their various dendritic spines, thereby increasing their processing and computational abilities as compared to other homologous neurons (Spruston, 2008). Even when considering human cortical layers and their encompassing cell types, the abundance of dendritic spines are significantly greater than seen in mice (Figures 3A–D) (Elston et al., 2001; Eyal et al., 2018; Iascone et al., 2020). A typical human cortical neuron is expected to demonstrate ~25,000–30,000 spines that possess larger spine heads, longer neck length and diverse morphologies than their rodent counterparts (Benavides-Piccione et al., 2002; Ofer et al., 2022). In comparison to mice, human cortical neurons form three to four times more synapses and have a higher ratio of asymmetric (excitatory) to symmetric (inhibitory) synapses per neuron in all layers except layer 4 (L4) (DeFelipe et al., 2002; DeFelipe, 2011; Benavides-Piccione et al., 2013). Overall, the unique morphological properties of human neurons with respect to size, dendritic length and arborization, projection targets, and number of synapses likely underscores the distinctive circuitry connections in humans that impart advanced cognitive and executive functioning (Galakhova et al., 2022). The enhanced memory capacity of human cortical neurons and their networks also suggests that these differences may have important implications for human learning and memory processing (Schmidt and Polleux, 2022).

### 3.2. Electrophysiological techniques demonstrating the functional specialization of human cortical neurons

The evolution of the human brain, particularly the increase in size and complexity, implies that there is a functional specialization in neuronal electrophysiological properties in the human cortex (DeFelipe, 2011). Human cortical pyramidal neurons exhibit an astonishing degree of electrophysiological heterogeneity within each cortical layer, which is consistent with transcriptomic and morphological heterogeneity (Deitcher et al., 1991; Zeng et al., 2012; Mohan et al., 2015; Kalmbach et al., 2018; Tasic et al., 2018; Berg et al., 2020; Moradi Chameh et al., 2021).

**Figure 2:**
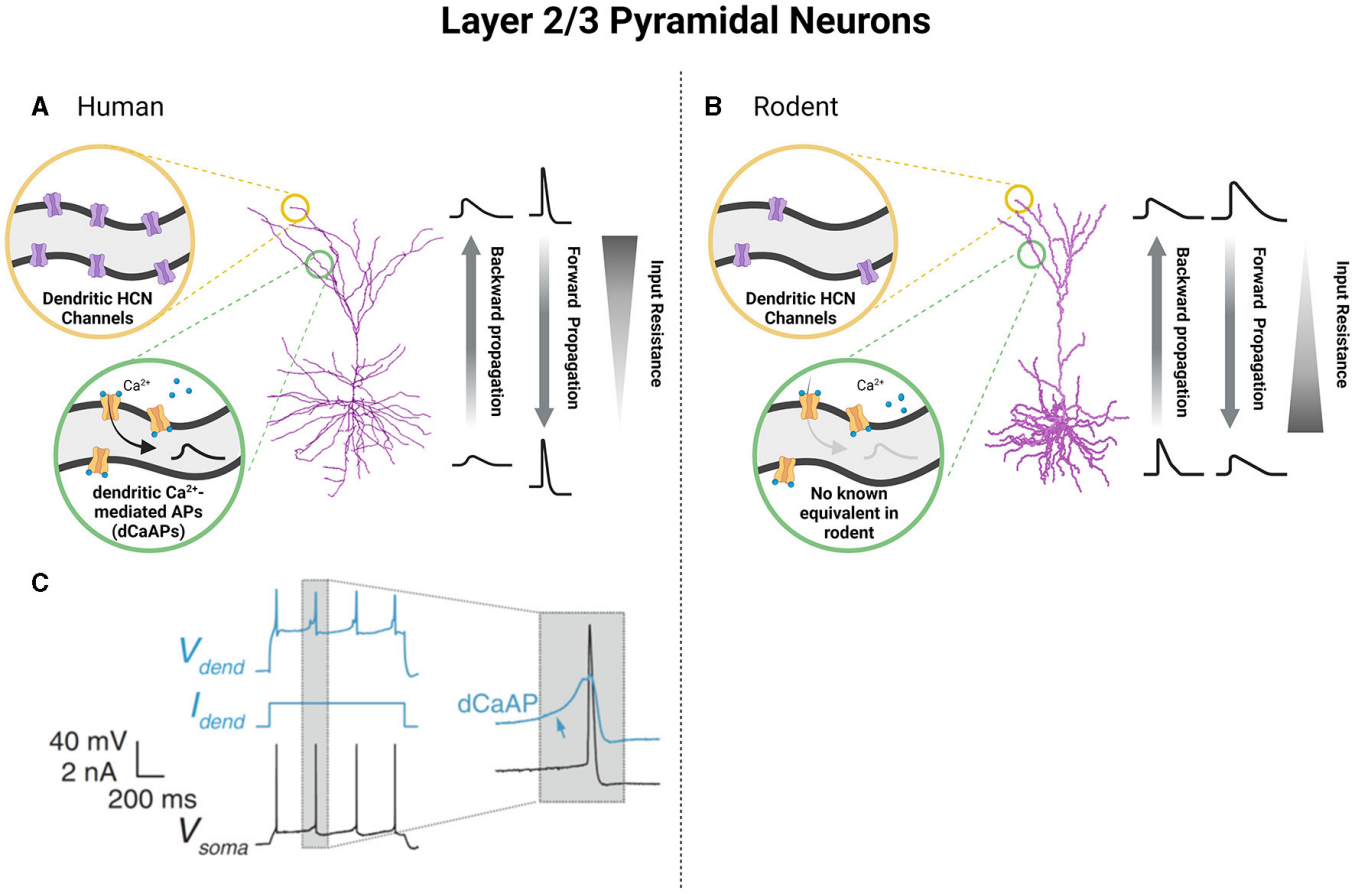
Unique functional specializations of cortical pyramidal neurons of L2 and L3 based on species-specific differences in neuronal biophysical properties. Pyramidal neurons of L2 and L3 of the human cortex show increased dendritic HCN channels **(A)** and the presence of dendritic calcium-mediated action potentials (dCaAPs) which work to reverse the attenuation of voltage that accompanies an increased dendritic length **(A, C)**. Alternatively, in L2/3 of the rodent cortex, pyramidal neurons show less dendritic HCN channels and no known equivalent of dCaAPs **(B)**. In turn, humans and rodent pyramidal neurons of L2/3 demonstrate differences in cable and active properties which alter the extent of backward and forward voltage propagation as well as input resistance values spanning neuronal length **(A, B)**. **(C)** was obtained from Gidon et al. (2020) and reprinted with permission from AAAS (License Number: 5601960942874). Created with BioRender.com.

The excitability of cortical pyramidal neurons in the L2 and L3 of the human cortex varies with depth and also between slim-tufted and profuse-tufted cells. Specifically, profuse-tufted cells have higher firing rate than slim-tufted cells (Deitcher et al., 1991; Kalmbach et al., 2018; Berg et al., 2020; Moradi Chameh et al., 2021) These biophysical differences allow these cells to process functionally independent information streams, with feedforward projections originating in L3 neurons and L2 neurons receiving feedback information (Markov and Kennedy, 2013). The differences in electrophysiological properties between human L2 and L3 neurons and rodent L2/3 neurons are likely functional manifestations of the increased dendritic length and arborization seen in humans (Deitcher et al., 1991; Mohan et al., 2015; Kalmbach et al., 2018; Berg et al., 2020; Moradi Chameh et al., 2021). As a result of the large size of human L2 and L3 neurons, these neurons undergo compartmentalization of the soma and dendrites that reduce electrical coupling between these areas. In turn, dendritic spiking does not necessarily yield somatic depolarization, which is commonly referred to as voltage attenuation (Beaulieu-Laroche et al., 2018; Gidon et al., 2020). To compensate for this compartmentalization and the resulting voltage attenuation, changes in membrane capacitance and channel expression are observed (Eyal et al., 2016; Kalmbach et al., 2018; Gidon et al., 2020). First, the membrane capacitance (Cm) of L2 and L3 pyramidal neurons in the human temporal cortex is approximately half of the commonly accepted value seen in rodent cortical neurons (~1 μF/cm^2^) (Eyal et al., 2016), however, see Beaulieu-Laroche et al. (2018). As a result, it is suggested that human L2 and L3 pyramidal neurons are able to transfer electrical signals along their dendritic arbors more efficiently, despite the increased length. Secondly, increases in the expression of hyperpolarization-activated cyclic nucleotide-gated (HCN) channels compensates for an attenuation of voltage through increasing HCN-mediated currents (Ih) and the reliability of transmission of electrical signals along long apical dendrites (Kalmbach et al., 2018). The expression of HCN channels in L2 and L3 of human cortical tissue demonstrates variability depending on depth, such that a higher expression of HCN channels is found in deep L3 (Kalmbach et al., 2018). This heterogeneous presentation of HCN currents is responsible for the functional specialization of neurons within these layers due to its role in altering input resistance, resting membrane potential, and sag voltage of neurons (Beaulieu-Laroche et al., 2018; Kalmbach et al., 2018; Moradi Chameh et al., 2021). Moreover, HCN channel expression and the consequent production of Ih is more prevalent in human cortical neurons than their rodent counterparts, indicative of species-dependent differences in neuronal function (Figures 2A, B) (Beaulieu-Laroche et al., 2018; Kalmbach et al., 2018). As an example, human pyramidal neurons in both deep L3 and L5 have a larger Ih than those in L2, which enhances their responsiveness to track the delta frequency compared to their rodent counterparts (Kalmbach et al., 2018; Moradi Chameh et al., 2021; Inibhunu et al., 2023). Finally, the attenuation of voltage in human cortical neurons is also compensated by the presence of calcium-mediated dendritic action potentials (dCaAPs) which act to enhance electrogenesis, the production of neuronal electrical activity, and excitability (Gidon et al., 2020) (Figures 2A, C). Overall, these alterations are unique to human cortical neurons for overcoming large dendritic distances.

**Figure 3:**
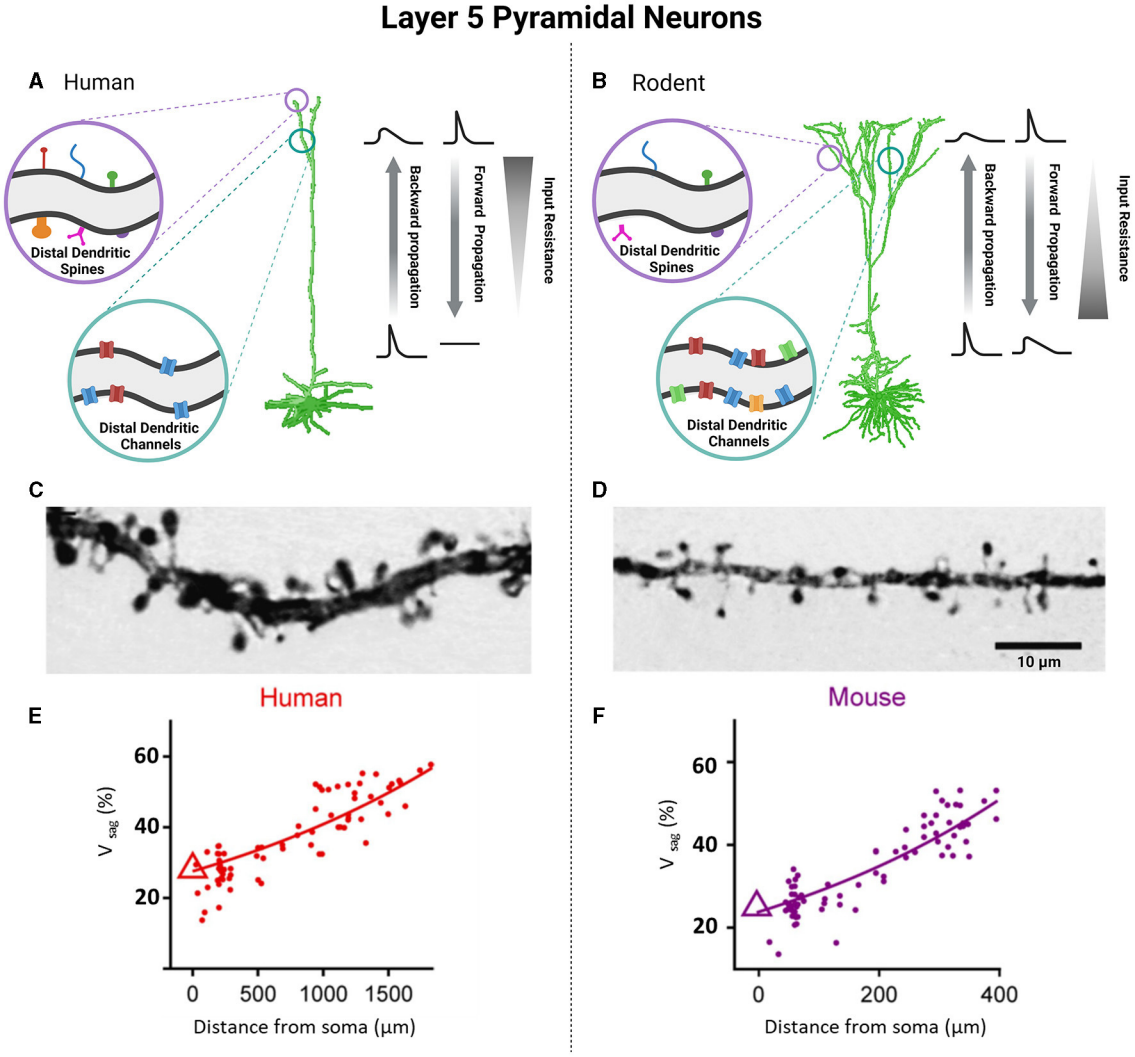
Unique functional specializations of cortical pyramidal neurons of L5 based on species-specific differences in neuronal biophysical properties. Pyramidal neurons of L5 between human and rodent cortices demonstrate notable differences as well. Human cells have an increased number and morphological diversity of dendritic spines **(A, C)**, but fewer dendritic channels compared to counterpart rodents **(B, D)**. Photomicrographs of L5 pyramidal neurons demonstrate this difference in density and size of dendritic spines in humans **(C)** vs. rodents **(D)**. The alterations of cable and active properties between human and rodent cells are responsible for differences in the backward and forward propagation as well as values of input resistance **(A, B)**. Voltage sag as a function of distance from the soma was greater in layer 5 pyramidal neurons of human **(E)** compared to mouse **(F)**. **(C, D)** were obtained from Benavides-Piccione et al. (2002) (License Number: 5601971059635). **(E, F)** were obtained from Beaulieu-Laroche et al. (2021) (License Number: 5601960704519). Created with BioRender.com.

Similar to rodent neurons, the dendrites of human cortical L2 and L3 pyramidal neurons are capable of actively propagating action potentials in both directions and generating local sodium/calcium spikes exhibiting both action potential backpropagation and locally generated sodium/calcium spikes (Beaulieu-Laroche et al., 2018; Gidon et al., 2020). However, the ability of human cortical L2 and L3 neurons to evoke NMDA spikes was found to be significantly reduced compared to that of rodent neurons, likely due to the larger diameter of the dendrites in humans (Testa-Silva et al., 2022). Unlike L2 and L3 of the human cortex, which showed remarkable differences from their rodent counterparts, L5 cortical cells of humans share some electrophysiological properties with mice (Beaulieu-Laroche et al., 2018; Kalmbach et al., 2021; Moradi Chameh et al., 2021). Specifically, there are two types of pyramidal neurons found in L5 of human and rodent cortical tissue: ET and IT cells. In both species, ET and IT cells show distinct electrophysiological features, including differences in membrane properties related to Ih such as sag voltage, rebound depolarization and rebound spike, resting membrane potential, and input resistance (Baker et al., 2018; Kalmbach et al., 2021). ET cells in both human and rodents exhibit larger sag voltage and rebound depolarization, depolarized resting membrane potential, and smaller input resistance compared to IT cells (Figures 3E, F) (Kalmbach et al., 2021). Due to their larger sag voltage, ET cells can track subthreshold resonance frequencies in the range of 3–6 Hz, while IT cells respond to lower frequency oscillations (<2.5 Hz) (Beaulieu-Laroche et al., 2018; Berg et al., 2021). The differential expression of HCN channels in ET cells plays a crucial role in the integration of synaptic inputs. ET cells, with their lower input resistance, exhibit lower firing rates compared to IT cells and display bursting activity near their threshold, which is likely to be associated with dendritic calcium electrogenesis, as previously reported (Kalmbach et al., 2021). However, a recent study showed that human L5 neurons have lower electrogenesis in distal dendrites compared with L5 neurons of other mammalian species, including rodents, rabbits, marmosets, and macaques, and thus increased computational compartmentalization (Beaulieu-Laroche et al., 2021). In terms of morphology, ET and IT cells exhibit similarities in both human and rodents. However, when it comes to electrophysiology, there are notable differences between ET cells in humans and rodents. ET cells in humans exhibit fast action potential (AP) kinetics and display high-frequency AP burst firing near threshold. This firing pattern is associated with dendritic calcium ion (Ca^2+^) electrogenesis (Kalmbach et al., 2021). Interestingly, the human IT cells can be further subdivided into IT-like 1 and IT-like 2. Human IT-like 2 cells exhibit a two-fold higher input resistance and larger rebound depolarization than IT-like 1 cells. Furthermore, the activity of IT-like 2 neurons is more similar to that of ET neurons, both exhibiting fast and narrow action potentials (Kalmbach et al., 2021).

In other words, each layer of the human cortex has a diverse repertoire of neurons that show unique functional specializations based on their electrophysiological features, underscoring the divergence between neuronal networks of human vs. preclinical animal models. These findings reflect species-related differences in neuronal physiology and emphasize the necessity to perform electrophysiological research on resected human tissue.

### 3.3. Transcriptomic techniques investigating the heterogeneity and laminar organization of the human cortical neurons

Recent breakthroughs in transcriptomic techniques, including the development of single cell/nucleus RNA sequencing (scRNA-seq/snRNA-seq) and patch-sequencing, have allowed researchers to quantify the RNA expression of hundreds to thousands of individual cells simultaneously (Zeng et al., 2012; Bakken et al., 2016; Cadwell et al., 2016; Lake et al., 2016; Wu et al., 2017; Tasic et al., 2018; Tripathy et al., 2018; Hodge et al., 2019). Notably, this field of research has set the foundation for investigating the transcriptomics of human neurons, discovering the extent of within-cell heterogeneity that is present in traditionally defined cell types and subtypes, and comparing these unique genetic profiles to other species (Lake et al., 2016; Hodge et al., 2019). Through assessing the transcriptome of human cortical tissue resected from patients with epilepsy or tumor, cell types representing non-neuronal, excitatory, and inhibitory cells can be classified based on molecular constituents. Keeping a reductionist mindset, each individual human cortical neuron can be assigned into a hierarchical classification system: (1) class, (2) subclass, (3), type, (4) and subtype. Transcriptomic techniques have shown that even within the narrowest classification of subtype, there is both continuous and discrete variation with respect to RNA expression (Zeng et al., 2012; Lake et al., 2016; Berg et al., 2021; Scala et al., 2021). Such variability is increasingly being accepted as a design feature of the brain rather than biological “noise” (Cembrowski and Menon, 2018; Cembrowski and Spruston, 2019), with recent work suggesting that biophysical diversity can stabilize brain dynamics in face of a number of “insults” (Rich et al., 2022; Hutt et al., 2023). The human MTG, which is the most heavily characterized region of the human brain electrophysiologically, demonstrates remarkable transcriptomic diversity in excitatory and inhibitory neuron classes, with 45 inhibitory and 24 excitatory cell types (Hodge et al., 2019). Interestingly, excitatory, and inhibitory neuronal cell types do not adhere to the tight laminar organization of the cortex and instead are found to be widely dispersed across the layers of the human cortex (Hodge et al., 2019). Due to the extent of heterogeneity within neuronal classes, subclasses, types, and subtypes, analyzing the transcriptome of individual cells rather than using laminar location represents a more reliable method for predicting the identity of a neuron (Zeng et al., 2012; Hodge et al., 2019).

Through comparisons of the transcriptomic homology between human MTG and rodent cortex, 21% of genes within the human cortex show species-specific differential expression from homologous areas in mice (Zeng et al., 2012). However, all major classes and subclasses of cortical neurons can be aligned between species on the basis of shared marker genes, suggesting highly conserved transcriptomic organization (Zeng et al., 2012; Hodge et al., 2019). This species conservation begins to deteriorate when examining gene expression at the cell type level which reveals a mix of convergent and divergent genes (Zeng et al., 2012; Hodge et al., 2019; Kalmbach et al., 2021). The considerable differences in transcriptomic profiles between corresponding cell types of human and rodent tissue demonstrates species-differences in cell type genetic profiles, proportions, laminar distribution, and even function (Zeng et al., 2012; Hodge et al., 2019). For example, human cortical L2 and L3 demonstrate notable differences in neuronal size, density, morphology, and electrophysiological properties that can be attributed to transcriptomic differences (Mohan et al., 2015; Lake et al., 2016; Kalmbach et al., 2018, 2021; Berg et al., 2021; Moradi Chameh et al., 2021). In contrast, neurons within L2/3 of the rodent cortex are homogenous, lacking the well-defined boundaries seen in the human such that the layers are often considered inseparable (DeFelipe, 2011; Markram et al., 2015). To some extent, these species-related differences in laminar organization occur as a result of five transcriptomic cell types- LTK, GLP2R, FREM3, CARM1P1 and COL22A1- being present in L2 (LTK, GLP2R) and L3 (FREM3, CARM1P1 and COL22A1) of the human cortex while only three homologous counterparts have been identified in L2/3 of the rodent. There is no homolog of CARM1P1 and COL22A1 in mice (Berg et al., 2021). Furthermore, gene expression patterns and markers that are characteristic of L5 in the rodent are either eliminated, reduced, or shifted to L3 of the human (Zeng et al., 2012). Overall, transcriptomic data has illuminated species-specific similarities and differences in laminar organization as well as neuronal cell types, thereby emphasizing the importance of conducting research on the human brain in addition to preclinical models (Hodge et al., 2019; Scala et al., 2021).

### 3.4. Functional specialization of human astrocytes

After myelin-competent oligodendrocytes, astrocytes comprise the largest class of glial cells in the mammalian CNS (Pelvig et al., 2008; Herculano-Houzel, 2014). They have key roles in maintaining the blood-brain barrier (Abbott et al., 2006), regulating regional blood flow (MacVicar and Newman, 2015), providing trophic, antioxidant, and metabolic support to neurons (Allaman et al., 2011), neurotransmitter uptake and recycling (Simonato et al., 2014; Weber and Barros, 2015), and regulating synaptogenesis and synaptic transmission (Araque et al., 2014; Chung et al., 2015). While these functions appear generally conserved across vertebrates, being equally documented in the murine and human brain (Vasile et al., 2017; Bedner et al., 2020), there is the emerging recognition of distinct human features for their cellular and molecular underpinnings (Oberheim et al., 2006; de Majo et al., 2020). As human neurons are larger than their rodent counterparts (Mohan et al., 2015), so are human astrocytes. Human astrocytes in the white matter have a maximum linear dimension of 183.2 μm, which is 2.1 times larger than the size observed in mice (85.6 μm) (Oberheim et al., 2009). Similarly, the extension of astrocyte branching domains in the human gray matter exhibits characteristic domain diameters about 2.6 times larger (142.6 vs. 56 μm), corresponding to a 16.5-fold greater occupied volume. The larger size of human astrocytes can be attributed to their more elongated and complex arborization, featuring 10 times more primary processes (37.5 vs. 3.8) that are, on average, 2.6-fold longer than mice (Oberheim et al., 2009). This increased complexity potentially reflects unique functional principles governing the organization of neural circuits in the human brain. Notably, a single astrocyte in the human brain can interact with millions of synapses, which is at least 10 times more than in mice (Bushong et al., 2002; Oberheim et al., 2009).

**Figure 4:**
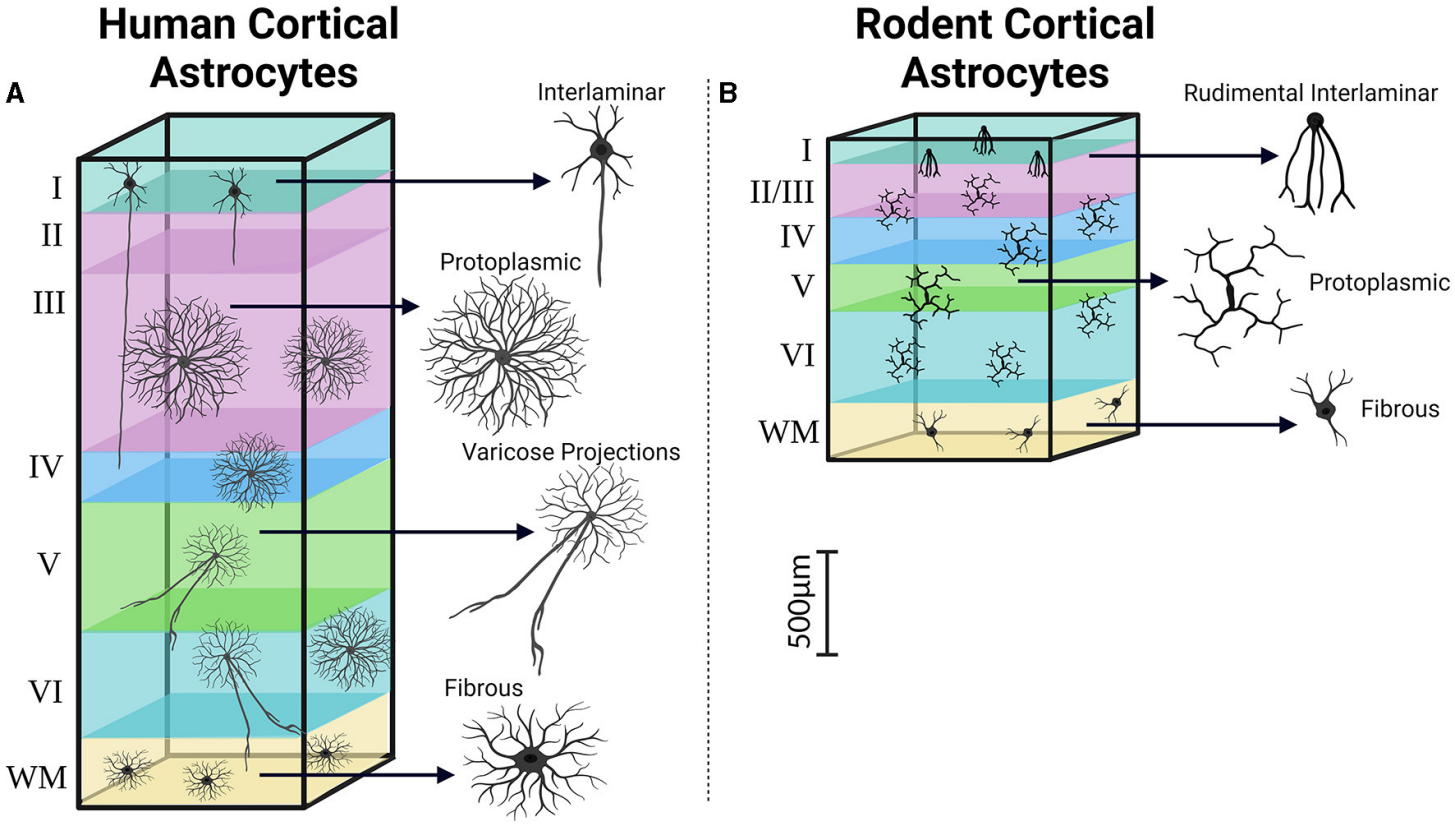
Cortical distribution and classification of astrocytes. Similar to the observations made regarding pyramidal neurons in the human and rodent cortex, astrocytes in the human cortex **(A)** demonstrate increases in size, complexity, and heterogeneity compared to their rodent counterparts **(B)**. Created with BioRender.com. WM, White matter.

Human astrocytes can be classified into four main categories based on their morphology. These categories include interlaminar astrocytes found in Layer I, protoplasmic astrocytes present in Layers I to VI, varicose projection astrocytes located in deep Layers V to VI, and fibrous astrocytes found in the white matter (Figure 4) (Colombo and Reisin, 2004; Oberheim et al., 2009; Shapson-Coe et al., 2021). Among these subtypes, varicose projection and interlaminar astrocytes seem bearing uniquely hominid features pinpointing to the existence of unique neuron-astrocyte circuits in Hominidae (Oberheim et al., 2012). Varicose projection astrocytes have indeed only been observed in humans and higher primates, differently from interlaminar cells, which could also be found in the murine cortex (Degl'Innocenti and Dell'Anno, 2023). Interlaminar astrocytes in humans and higher primates however show increased complexity in terms of higher number of branches, with multiple processes infiltrating deeper cortical layers as opposed to the rudimental anatomy of their murine counterpart which are instead generally contained within their layer of origin (Colombo and Reisin, 2004; Falcone et al., 2021). Furthermore, the positional arrangement of astrocytes in the human cortex is likely to have distinct characteristics. Ultra-resolution microscopy of nonepileptic cortical tissue obtained from MTLE patients supports this notion revealing densely packed aggregates of intermingling protoplasmic astrocytes throughout all cortical layers (Shapson-Coe et al., 2021). This finding contrasts sharply with the observation that astrocyte domains are minimally overlapping in the mouse brain instead (Bushong et al., 2002; Ogata and Kosaka, 2002; Abdeladim et al., 2019).

Our understanding of the biophysical basis for the morphological specialization of human neuron-astrocyte circuits is still in its infancy. The notable size difference between human and rodent astrocytes explains the larger capacitance of the former (44.7 pF vs. ~10 pF−25 pF) (Bordey and Sontheimer, 1998; Amzica and Neckelmann, 1999). However, the membrane resistance of human astrocytes can range from values as low as 4.5 ± 3 MΩ (Bedner et al., 2020), similar to that observed in rodents, to 64-fold higher values (288 MΩ) (Bordey and Sontheimer, 1998). This suggests that the molecular composition of human astrocyte's membranes could differ substantially from that of mice. There may also be significant differences in intracellular properties. One crucial intracellular signal in astrocyte physiology is calcium (Ca^2+^), which plays a role in ion homeostasis, synaptic activity sensing, and its modulation (Navarrete et al., 2012; De Pittà et al., 2015; Semyanov et al., 2020). Interestingly, several genes associated with Ca^2+^ homeostasis, namely RYR3, MRVI1, and RGN, were found to be more abundant in human astrocytes than in mice (Zhang et al., 2016). Furthermore, the average propagation velocity of Ca^2+^ signals through astrocyte networks is considerably higher in humans (43.4 μm/s) compared to their murine counterparts (8.6 μm/s) (Oberheim et al., 2009).

Another noteworthy example is the co-expression of GDH1 and GDH2 genes in human astrocytes but not in rodents (Zhang et al., 2016). These genes encode glutamate dehydrogenase, which converts glutamate into α-ketoglutarate as the first step in the GABA/glutamate-glutamine cycle underpinning neurotransmitter recycling at central inhibitory and excitatory synapses (McKenna et al., 2016). Accordingly, it was shown that GDH1 and GDH2 co-expression enables human astrocytes to better withstand increased metabolic demands (Nissen et al., 2017), such as those arguably arising from their extensive synaptic interactions. Altogether, these findings indicate that human astrocyte diversity involves unique molecular characteristics, such as distinct Ca^2+^ signaling and enhanced metabolic capabilities, which may contribute to the functional complexity of the human brain.

Recent advancements in transcriptomics have provided unparalleled insights into the molecular characteristics of human astrocytes. Genetically, human astrocytes exhibit a distinct set of expressed genes that are either absent or significantly reduced in rodents (Miller et al., 2010; Zhang et al., 2016). Some of these genes' expression strongly correlates with the morphological features observed in human astrocytes. One such gene is PMP2, which encodes a cytoplasmic fatty-acid binding protein that appears to contribute to the larger size of human astrocytes (Kelley et al., 2018). Another example is CD44, which encodes a cell-surface glycoprotein involved in cell-cell interactions, cell adhesion, and migration. CD44 is prominently expressed by varicose projection and intralaminar astrocytes, potentially explaining their morphological signature characterized by long unbranched processes that extend across adjacent astrocytes (Sosunov et al., 2014). Human astrocytes' spatial location and transcriptional identity are determined by their embryonic site of origin, independent of neurons (de Majo et al., 2020). Interestingly, cultures of human embryonic stem cells can be patterned to generate astrocytes with specific regional identities even without inductive cues from neurons (Krencik et al., 2011). This could be explained by the existence of human-specific astrocyte progenitors that might account for uniquely human astrocyte populations (Hansen et al., 2010; Pollen et al., 2015), although the mechanisms of differentiation of astrocyte lineages from single progenitors remain to be explored (Beattie and Hippenmeyer, 2017; Ostrem et al., 2017). On the other hand, astrocytes, in the presence of multiple extracellular signals originating from the surrounding neuropil, can undergo a wide range of intracellular and transcriptional changes that can influence or maintain their heterogeneity (Haim and Rowitch, 2017). Correlational analysis of RNA-seq data from numerous post-mortem human brain specimens supports this scenario, revealing that, in addition to the presence of unique genes, astrocyte transcripts co-vary with the cellular composition of the brain area they live in (Kelley et al., 2018).

Besides anatomic distribution, age and pathology are crucial in determining astrocyte transcriptional and functional identity. In line with the observed limited response of the white matter to injury in fetal brains, human fetal astrocytes exhibit significantly lower expression levels of pro- and anti-inflammatory microRNAs (MiRs) compared to adult human astrocytes (Rao et al., 2016). Similarly, MiR-124, the regulator of the type 2 excitatory amino acid transporter (EAAT2), shows low or undetectable levels in fetal astrocytes but is abundant in adult astrocytes (Fine et al., 1996; Liang et al., 2002; Rao et al., 2016). These findings align with the understanding that EAAT2 is predominantly functionally expressed by mature cells (Sloan et al., 2017). The onset of pathology can elicit a wide range of molecular, transcriptional, and morphological changes in astrocytes, collectively referred to as reactive astrogliosis (Sofroniew, 2015; Escartin et al., 2021). However, the extent to which astrocyte functional diversity is maintained under pathological conditions remains unclear, as both toxic gains and loss of function have been proposed (Liddelow and Sofroniew, 2019). For instance, the decreased functional expression of the astrocytic enzyme glutamine synthetase (GS) in patients with MTLE has long been associated with elevated extracellular glutamate levels in this condition. The loss of GS activity slows down the conversion of glutamate to glutamine in astrocytes, contributing to the extracellular accumulation of glutamate (Eid et al., 2004). However, recent experiments have revealed that this loss of GS expression may not correlate with astrocyte reactivity. Instead, it may result from subcellular down-regulation or redistribution of GS while still maintaining detectable regional GS expression (Papageorgiou et al., 2018). This evidence supports the idea that, beyond inherited transcriptional factors, human astrocyte identity and functional specialization is ultimately tightly regulated by a diverse set of intrinsic and extrinsic factors within the specific neural circuits they inhabit (Belin et al., 1997; Eid et al., 2013; Haim and Rowitch, 2017).

## 4. Human-specific differences in synaptic properties and collective dynamics

**Figure 5:**
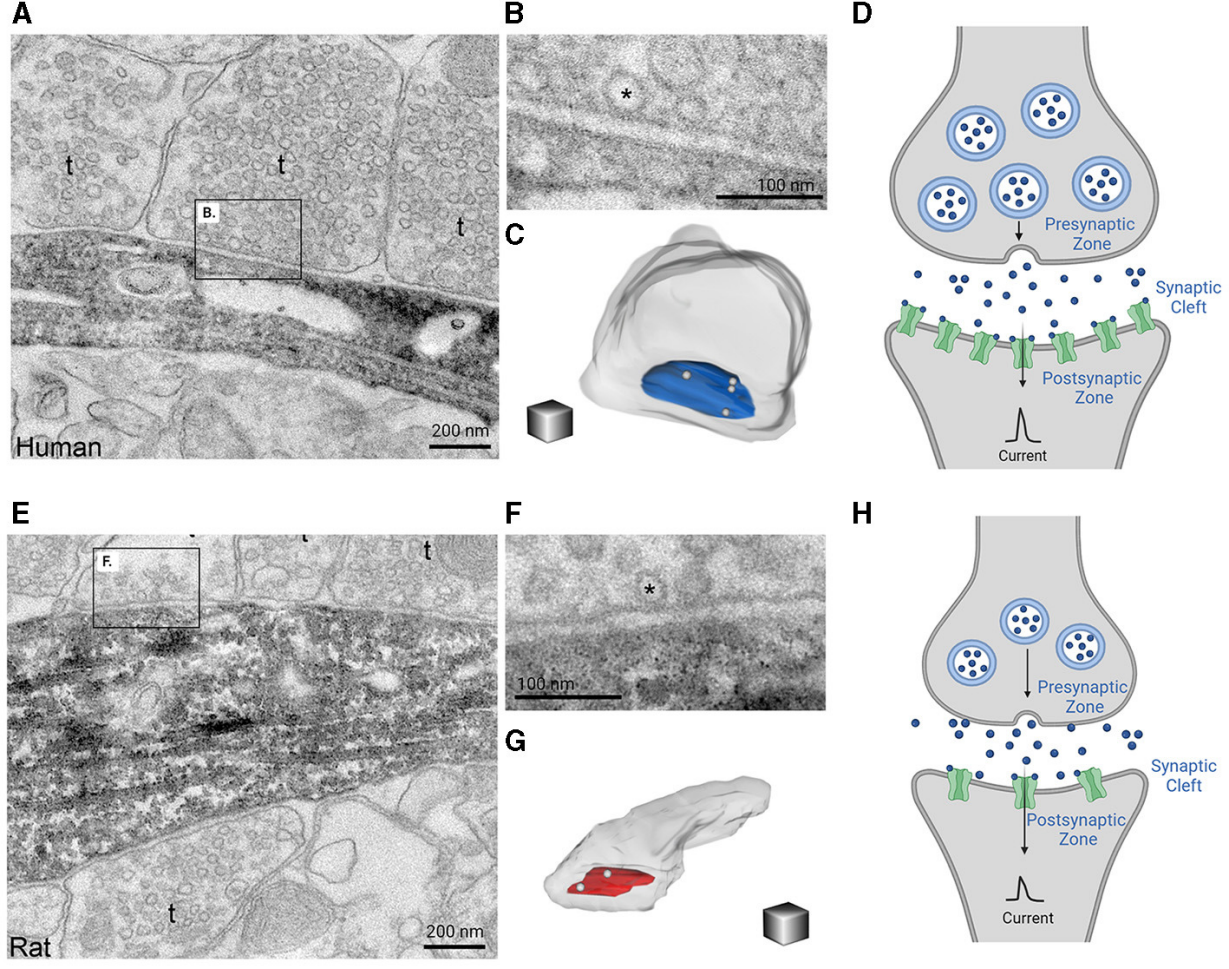
Glutamate synaptic morphology in human and rodent cortices lay the foundation for species-specific differences in information processing. Electron microscopy images of 20 nm thick sections reveals that human synapses are larger in size **(A)** and have increased docking of vesicle pools **(B)** when compared to rat synapses **(E, F)**. Further 3D reconstructions of synapses demonstrates that human synapses also have a larger presynaptic active zone (pictured in blue) compared to rats (pictured in red) **(C, G)**. This information is summarized in **(D, H)** which demonstrate that human synapses **(D)** are overall larger in size with increased vesicle pools, presynaptic active zones, and density of postsynaptic channels as compared to rodents **(H)**. The consequent production of synaptic currents between human cortical neurons is stronger and larger. The electron microscopic images of 20 nm thick sections of the axon terminals and synapse for a human and rat as well as their 3D reconstructions were obtained from the eLife publication by Molnár et al. (2016). Inclusion of these figures falls under the Creative Commons Attribution 4.0 International Public License (https://creativecommons.org/licenses/by/4.0/legalcode). Created with BioRender.com.

Human cortical circuits demonstrate distinctive signatures of collective activity, arising not only from specific biophysical features of human cortical neurons, but also from their unique functional connectivity to one another. Some of the first evidence identifying the unique network properties of human cortical circuits came from quadruple patch recordings that revealed long lasting polysynaptic events in human cortical circuits could be elicited with a single presynaptic action potential, that were an order of magnitude longer than observed in other species (Molnár et al., 2008). These polysynaptic events arose from a unique sequence of events that involved axo-axonic interneurons generating a third order spike in pyramidal neurons. In part these reverberant activities have been attributed to the larger presynaptic active zones, postsynaptic densities, and vesicle pools of pyramidal neurons from the MTG than in mice (Figures 5A–H) (Benavides-Piccione et al., 2002; Molnár et al., 2016; Yakoubi et al., 2019). These findings reflect the stronger and more reliable synapses that are present in the human cortex (Hunt et al., 2022). Moreover, human cortical synapses are not only stronger and have larger synaptic currents in individual synaptic connections as compared to rodents, but also demonstrate increased resilience to repeated activation (Testa-Silva et al., 2014). The overall increased size of human cortical synapses, seen in Figure 5A, may be attributed to compensatory mechanisms that work to overcome characteristics of human neurons such as dendritic length and potential voltage attenuation, as discussed earlier (Beaulieu-Laroche et al., 2018; Gidon et al., 2020; Kalmbach et al., 2021) (Figures 2, 3). Aside from being larger, excitatory synapses of the human MTG pyramidal neurons form more depressing synapses with three times faster recovery times than those observed in rodents which allow neurons to distinguish fast incoming inputs increasing the temporal resolution of human synapses (Testa-Silva et al., 2014). As a result, human cortical neurons are able to track inputs at higher rates and encode nine times more information at a higher bandwidth than than rodents (Testa-Silva et al., 2014). Moreover, the window of spike-timing dependent plasticity is wider in human cortical neurons, which has many important functional implications on activity-dependent synaptic modifications including the consolidation of transient effects into long-lasting circuitry changes (Schmuhl-Giesen et al., 1991; Yakoubi et al., 1991, 2019).

These unique aspects of synaptic physiology coupled with human specific biophysical properties (Moradi Chameh et al., 2021; Rich et al., 2021; Inibhunu et al., 2023) may underlie the emergence of *in-vitro* theta oscillations, which in general are not elicited in *in-vitro* rodent cortical slices, yet are the most ubiquitous oscillations in the human brain (Groppe et al., 2013). Human cortical slices have been shown to generate theta oscillations when exposed to kainic acid and carbachol (a cholinergic agonist) (Florez et al., 2013; McGinn and Valiante, 2014) which resemble theta oscillations *in-vivo* (Rizzuto et al., 2003; Herweg et al., 2020). Notably both *in-vivo* and *in-vitro* (Florez et al., 2013) theta oscillations demonstrate stable frequency dynamics and phase-amplitude coupling to high-frequency activity (Canolty et al., 2006; McGinn and Valiante, 2014). In addition to the higher probability of spiking propagation in human cortical circuits, unique biophysical properties of human L5 pyramidal neurons likely facilitate spiking at theta frequencies. Likely due to the differences in the h-channel kinetics of human Ih human L5 neurons do not demonstrate a subthreshold resonant peak (Moradi Chameh et al., 2021; Rich et al., 2021) in the low theta range that is observed in rodents (Silva et al., 1991; Lampl and Yarom, 1997; Schmidt et al., 2017). However, characterization of the frequency dependent gain of human L5 pyramidal neurons, has demonstrated a clear spiking preference at delta and theta frequencies, suggesting a double “tuning” of human L5 pyramidal neurons, allowing them to amplify both delta and theta frequency activity (Inibhunu et al., 2023). Such tuning is likely to underlie the ubiquity of theta oscillations (Groppe et al., 2013), since L5 pyramidal neurons play a critical role in setting the excitability of the entire cortical column (Beltramo et al., 2013), and likely drive the coherent oscillations seen in human cortical slices (McGinn and Valiante, 2014).

## 5. Within-cell type heterogeneity in the context of human neurosurgical tissue

A critical question regarding the cellular electrophysiology of human neurons from epilepsy patients, is whether or not the measured means of the biophysical parameters reflect normality. Here, we reviewed that the proximity of pyramidal neurons to the epileptogenic focus demonstrates more morphological and synaptic changes as well as increased susceptibility to electrical and convulsant chemicals. However, when examining the intrinsic biophysical parameters derived from epilepsy vs. control conditions, where the control tissue consists of healthy, non-epileptogenic neurons from tumor patients, there is no appreciable difference. In turn, we here present another line of evidence to support the notion that means of biophysical parameters of human cortical neurons, particularly those from the MTG likely reflect normalcy. Below we introduce the concept of biophysical diversity as represented by the population variance of a or multiple within-cell type biophysical features. We argue that the decrease in variability can explain how in light of “normal” mean parameter values pathological dynamics arise for decreases in their standard deviation.

An often-overlooked aspect of the biophysical features of neurons is their variance within a cell-type (Santhakumar and Soltesz, 2004). Recent RNA-seq and patch-seq studies have reinforced that transcriptomic and electrophysiological variability of given cell-type is an important lens through which to understand cellular diversity (Tasic et al., 2016; Scala et al., 2021), and that such variability, beyond biological noise, is an important design feature of the brain (Cembrowski and Menon, 2018). Computational neuroscience for a long time has highlighted how variability in biophysical features among neurons in a circuit increases information coding (Tripathy et al., 2013; Rich et al., 2022; Hutt et al., 2023). We have more recently shown that human epilepsy is accompanied by a loss of biophysical diversity (i.e., neural homogenization) which renders simulated circuits unstable and prone to sudden transitions to seizure-like activity (Rich et al., 2022). Our related theoretical work has shown how excitability heterogeneity enriches the dynamics of cortical circuits while rendering them resilient to destabilizing insults (Hutt et al., 2023). A key finding from our experimental work was that L5 pyramidal neurons of seizure prone cortex demonstrated neuronal homogenization in excitability, without a change in their mean values. Similarly, in a preclinical rodent model of epilepsy only the variance but not the mean of the resting membrane potential of stratum oriens interneurons was altered during epileptogenesis (Aradi and Soltesz, 2002).

**Figure 6:**
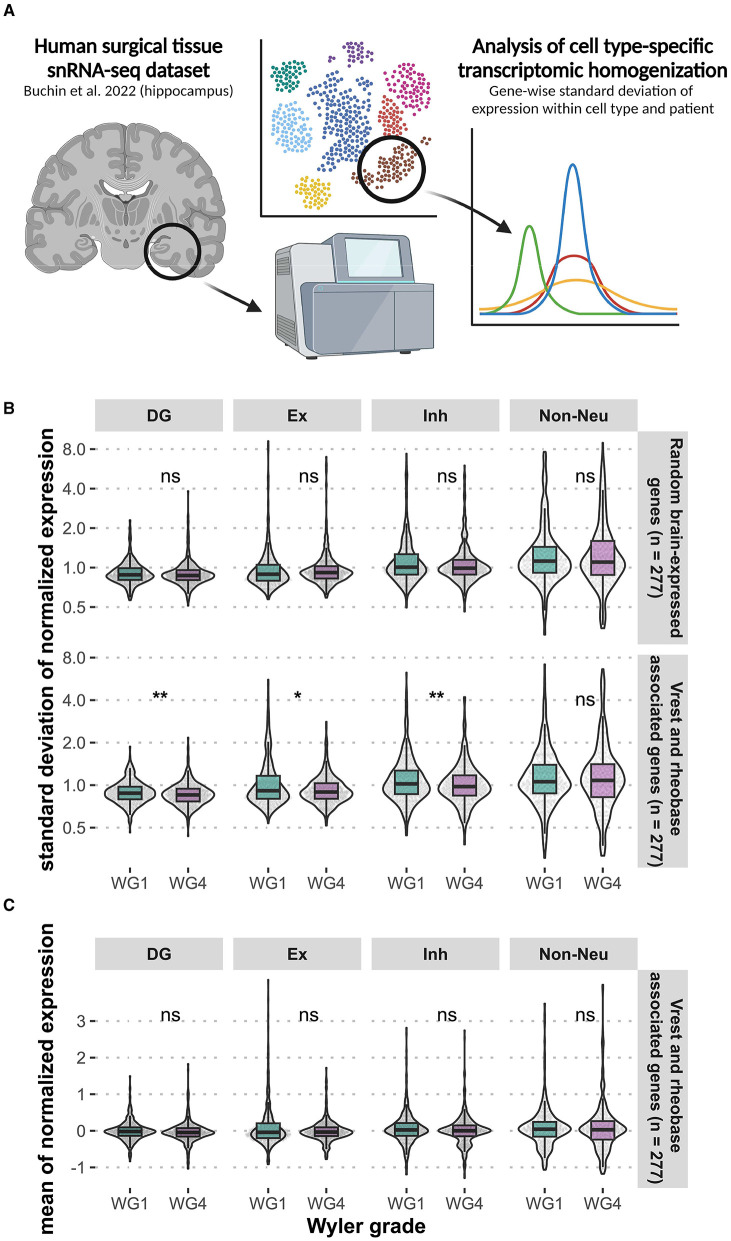
Loss of within-cell type transcriptomic heterogeneity in genes associated with intrinsic neuronal properties of the human dentate gyrus. We examined the transcriptomic heterogeneity of genes associated with intrinsic neuronal properties, such as resting membrane potential (Vrest) and action potential threshold (rheobase) **(A)**. Analysis of existing hippocampal snRNA-seq data (Buchin et al., 2022) from epilepsy patients revealed significant reduction standard deviation of gene expression (*p < 0.05, **p < 0.01, Wilcoxon) across neuronal cell types in tissue with a greater degree of epilepsy pathology (Wyler Grade 4) **(B)**. The same differences were not observed for mean normalized expression **(C)**. DG, dentate gyrus granule cells; Ex, excitatory (non-DG granule cells); Inh, inhibitory. Created with BioRender.com.

Our physiology data from the resected human neurosurgical tissue suggests that such epilepsy-driven neural homogenization might occur via intrinsic plasticity mechanisms (Zhang and Linden, 2003; Beck and Yaari, 2008), driven by transcriptomic homogenization. To examine this, we made use of a unique dataset of single-nucleus gene expression profiles from dentate gyrus granule cells and other neurons from sclerotic human hippocampus collected from neurosurgical resections from human patients with different grades of epilepsy pathology (Figure 6A) (Buchin et al., 2022). Using these data, we investigated between-group variability in transcript expression levels of genes that we have previously bioinformatically associated with intrinsic neuronal properties, such as resting membrane potential (RMP) and action potential threshold (Tripathy et al., 2017) (Figure 6A). For all genes, we calculated the standard deviation of normalized expression independently within each cell type and patient, reasoning that lower standard deviations indicate greater loss of within-cell type heterogeneity. We observed significant reductions in the standard deviation of gene expression of gyrus granule cells and other neurons from sclerotic human hippocampus (Figure 6B) (Buchin et al., 2022), where epilepsy patients with a greater degree of epilepsy pathology (Wyler Grade 4) also demonstrated a greater degree of transcriptomic homogenization relative to patients with more mild pathology (Wyler Grade 1). Importantly, differences in standard deviation were observed in the absence of differences in mean gene expression (Figure 6C). Additional single cell datasets of human tissue are needed to further assess the relationship between transcriptional heterogeneity and epilepsy.

An additional potential mechanism for neural homogenization is through ion channel degeneracy (Goaillard and Marder, 2021; Stöber et al., 2023), whereby biophysical homogenization could arise via a myriad of ion channels expression level changes while rendering neurons similar biophysical (Goaillard and Marder, 2021). Indeed, in face of environmental insults, lobster stomatogastric ganglion neurons maintain their designated function through profound changes in ion channel expression levels (Goaillard and Marder, 2021). We propose that epileptogenesis might be such an insult that increases biophysically homogeneity rendering circuits unstable (Aradi and Soltesz, 2002; Lengler et al., 2013; Rich et al., 2022; Hutt et al., 2023), without a change in mean values. Although the mechanism by which neural homogenization occurs in epileptogenesis remains to be elucidated we have observed that the mean values of neuronal properties change very little if at all through the process (Aradi and Soltesz, 2002; Rich et al., 2022). In this light then, we propose that the values of biophysical features of neurons from tissue epilepsy patients are abnormal not in their means but in the variances. While the decreased variances are likely pathological (Rich et al., 2022; Hutt et al., 2023), the mean values likely reflect normalcy.

This conclusion is further strengthened by orthogonal data obtained from studies in tumor patients without epilepsy, who show no difference in the mean values of biophysical parameters between epilepsy and control groups (Deitcher et al., 1991; Mohan et al., 2015; Kalmbach et al., 2018; Rich et al., 2022). There is no evidence to indicate that the etiology of the patient's disease, epilepsy or tumor, is correlated with morphological, electrophysiological, and transcriptomic properties of the tissue resected from the MTG (Mohan et al., 2015; Kalmbach et al., 2018). Furthermore, various studies have found the mean values of biophysical and synaptic properties of human penumbral tissue (MTG) remain insignificantly different from those of control groups including “normal” cortical tissue removed during tumor resections or homologous tissue from animals (rodents or macaque monkeys) (Schwartzkroin and Prince, 1976; Avoli, 1993; Mohan et al., 2015).

In summary, the above observations help to reconcile the original work in human cortical tissue where the *in vitro* search for the “epileptic neuron” failed (Ward, 2010). In light of the human cortical studies reviewed above, neural homogenization is enough to destabilize networks toward seizure-like states, without observed changes in mean parameter values.

## 6. Conclusion

The advent of investigations using resected brain tissue from humans has advanced both neurosciences, and more specifically the study of human brain mechanisms. Through the characterization of human neuronal biophysical properties, the intra- and inter-species differences that exist relative to commonly employed preclinical animal models are being addressed. Furthermore, by reviewing various morphological, electrophysiological, and transcriptomic studies, we have emphasized the significant species-related differences that exist between neuronal structure and functioning which necessitate the importance of conducting research on the human brain in addition to preclinical animal models. Finally, we suggest that within-cell type heterogeneity in morphological, electrophysiological, and transcriptomic features of neurons is a design feature conserved across species, with losses of such diversity able to explain pathological brain activity despite unaltered mean values.

## Author contributions

HMC contributed to the content and structure, conducted a literature search to acquire relevant information, wrote the initial draft and graphic design. MF contributed to the manuscript by writing, editing, and graphic design. MM contributed to the graphic design and editing. KA and SJT contributed to data analysis and graphic design for Figure 6, and editing. SR, LZ, and JL contributed to editing. MDP contributed to writing of the astrocytes section. TV contributed to the writing, editing, content and structure, and accuracy of the document's descriptions throughout. All authors contributed to the article and approved the submitted version.
